# Effects of sterilization methods on the survival of pathogenic bacteria in potting soil stored at various temperatures

**DOI:** 10.1007/s10068-022-01173-1

**Published:** 2022-11-22

**Authors:** Jeong-Eun Hyun, Su-Bin Lee, Do-Young Jung, Se-Ri Kim, Song-Yi Choi, Injun Hwang

**Affiliations:** 1 Microbial Safety Division, Department of Agro-Food Safety and Crop Protection, National Institute of Agricultural Sciences, Rural Development Administration, 166, Nongsaengmyeong-ro, Wanju-gun, Jellabuk-do 55365 Republic of Korea; 2 Department of Environmental Science and Biotechnology and Food, Jeonju University, 303, Cheonjam-ro, Jeonju-si, Jellabuk-do 55069 Republic of Korea

**Keywords:** Pathogenic bacteria, Survival, Potting soil, Sterilization method, Ginseng sprouts

## Abstract

Fresh food products can be contaminated with pathogenic bacteria in various agricultural environments. Potting soil is sterilized by heat sterilization and then reused. This study evaluated the effects of three sterilization methods (non-sterilized, pasteurized, and sterilized) on the survival of pathogenic bacteria in potting soil during storage for 60 days at 5, 15, 25, and 35 °C. The reduction in *Escherichia coli* O157:H7, *Salmonella* Typhimurium, and *Staphylococcus aureus* in potting soil was higher at higher temperatures (25 and 35 °C) than at lower temperatures (5 and 15 °C). The population of pathogenic bacteria in pasteurized and sterilized potting soil was reduced below the detectable levels within 30 days at 35 °C. In contrast, the population of *Bacillus cereus* did not change in potting soil during storage for 60 days at all temperatures. These results indicate that sterilization and storage temperature of potting soil are critical factors influencing the survival of pathogenic bacteria.

## Introduction

Fresh, nutritious, and healthy foods such as vegetable sprouts, which are usually consumed raw, can be contaminated by various microorganisms. From 2005 to 2017, *Escherichia coli* O157:H7 and *Salmonella* spp. were the major pathogenic bacteria contributing to outbreaks in alfalfa sprouts (Centers for Disease Control and Prevention, 2016), clover sprouts (CDC, 2017), and mung bean sprouts (Rohekar et al., 2008). In 2011, a large outbreak of *E. coli* O104:H4, which was associated with the consumption of raw vegetable sprouts, occurred in Europe (European Food Safety Authority, 2011). In general, vegetable sprouts can be contaminated with pathogenic bacteria in various agricultural environments during harvesting. Possible sources of contamination in agricultural products are soil, raw manure, compost, irrigation water, and human handling (Olaimat and Holley, 2012). Although soil provides essential nutrients for agricultural product growth, improper management can be attributed to a major source of contamination during seed germination (Steele and Odumeru, 2004).

Ginseng spouts (*Panax ginseng* sprouts) are grown in greenhouses using soil-based or hydroponic systems at 23–24 °C and harvested after approximately 25–40 days (Chang et al., 2020). Among the types of soil, potting soil, also known as growing media or cultural media, is an active substance that supplies nutrients and moisture required for growth in ginseng sprouts. Most of potting soil is reused without sterilization or disinfection until an injury occurs by the continuous cropping of ginseng sprouts. Hyun et al. (2022) reported populations of *Bacillus cereus* ranged from 1.00 to 4.84 log CFU/g on 20 ginseng sprouts distributed in Korea. A previous study showed that the levels of total aerobic bacteria differed depending on the site of ginseng sprouts, with average populations of 5.69, 5.17, and 7.15 log CFU/g in the leaves, stems, and roots of ginseng sprouts, respectively (unpublished data). These results indicated a higher level of contamination in the roots than in the leaves and stems. The survival and growth of pathogenic bacteria in soil are determined by various factors, such as light, pH, moisture content, temperature, and soil component (Alegbeleye et al., 2018). Several studies have reported that *E. coli* O157:H7 and *Salmonella* spp. can survive in the soil from 7 up to 25 weeks, depending on the temperature, moisture content, and type of soil (Guo et al., 2002; Lang and Smith, 2007; Nicholson et al., 2005; Zhang et al., 2009). Solomon et al. (2002) also demonstrated that *E. coli* O157:H7 was detected in lettuce cultivated on soil using manure or irrigation water contaminated with *E. coli* O157:H7. According to these results, pathogenic bacteria can enter the roots of agricultural products and thus increase contamination levels of pathogenic bacteria in agricultural products. Thus, there is a need to develop efficient technologies to control pathogenic bacteria and improve soil safety. Various sterilization technologies, including heat treatment using autoclaving, γ-radiation, and ethylene oxide, have been used to change the organic matter in the soil (Berns et al., 2008; McNamara et al., 2003; Nègre et al., 1995). However, the effect of sterilization methods on the survival of pathogenic bacteria in potting soils has not been intensively studied. Therefore, this study aimed to investigate the survival of pathogenic bacteria in potting soil using different sterilization methods (non-sterilized, pasteurized, and sterilized) during storage at various temperatures (5, 15, 25, and 35 °C).

## Materials and methods

### Bacterial strain and culture conditions

Three strains of *E. coli* O157:H7 (ATCC 13890, 43889, and 43894), *Salmonella * Typhimurium (ATCC 19586, 43174, and DT104), *Staphylococcus aureus* (ATCC 13565, 23235, and 25923), and *Bacillus cereus* (ATCC 13061, KACC 1092, and KACC 13066) were obtained from the Bacterial Culture Collection of the National Institute of Agricultural Sciences (Wanju-gun, Jeollabuk-do, Korea). Each strain was cultured in tryptic soy broth (TSB; Difco Laboratories, Detroit, MI, USA) at 37 °C for 24 h before use. Antibiotic-marked strains were used because microorganisms are naturally present in the soil. The rifampicin-resistant strain was prepared as previously described (Choi et al., 2017) with some modifications. A stock solution of 1 mg/mL (w/v) rifampicin (Gold Biotechnology, St. Louis, MO, USA) was prepared in dimethyl sulfoxide (Biosesang, Seongnam-si, Gyeonggi-do, Korea). The solutions were filtered through a 0.45 μm syringe filter (Taeshin Bio Science, Namyangju-si, Gyeonggi-do, Korea). Each strain was inoculated with TSB containing 50 μg/mL rifampicin and incubated at 37 °C for 24 h. Subsequently, each strain was spread on tryptic soy agar (TSA; Difco) containing 50 μg/mL rifampicin. All rifampicin-resistant strains were cultured in TSB containing 50 μg/mL rifampicin at 37 °C for 24 h, harvested by centrifugation at 6000 × *g* for 5 min at 4 °C, and washed twice with sterilized 0.1 M phosphate-buffered saline (PBS, Biosesang). The cell pellets were resuspended in 0.1 M PBS to prepare a bacterial culture with a concentration of approximately 10^7^–10^8^ CFU/mL. For cocktail preparation, equal volumes of the three strains of pathogenic bacteria were mixed (1:1:1, v/v/v), and the cell suspension was used as an inoculum.

### Preparation of potting soil and inoculation

**Fig. 1:**
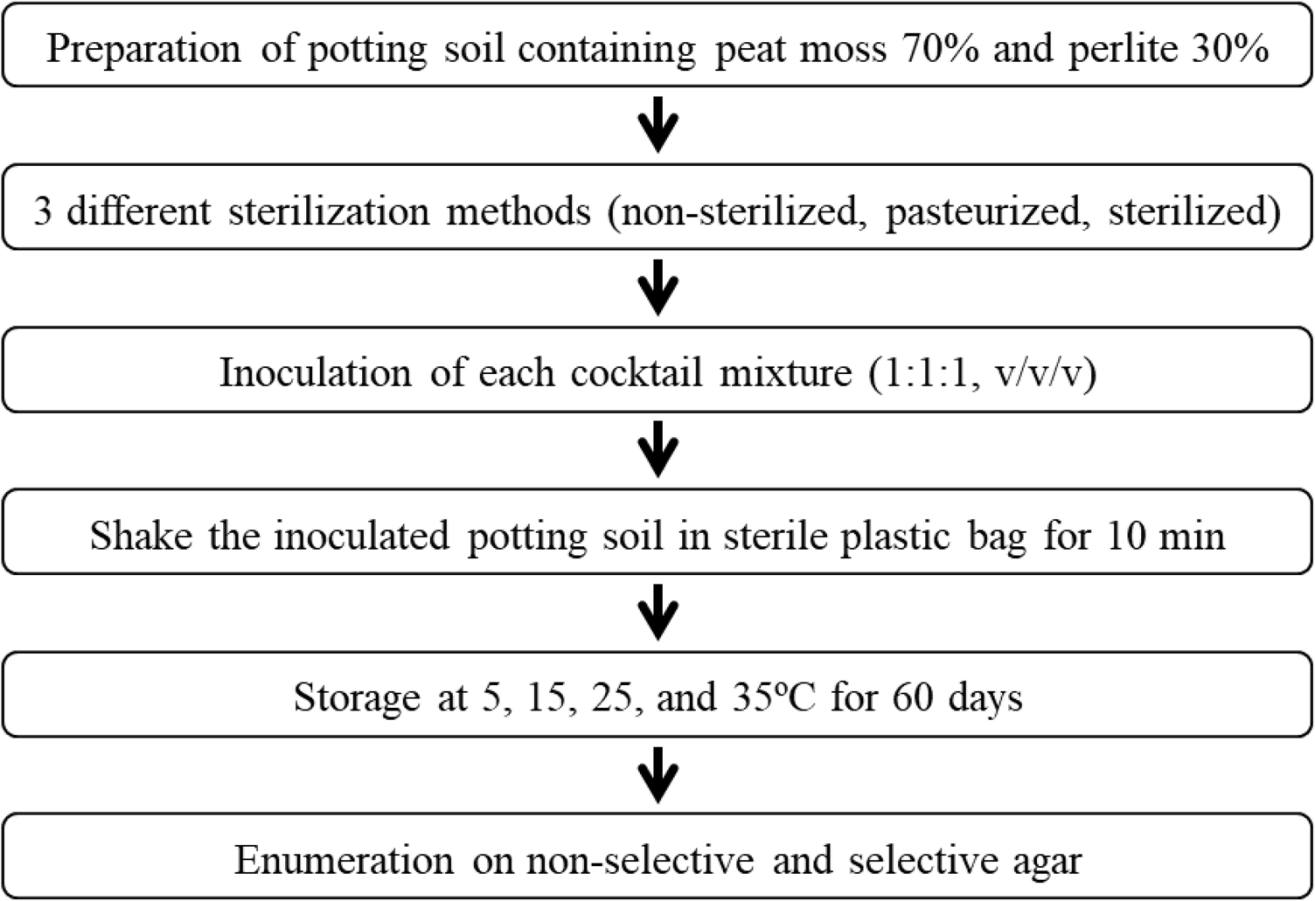
Flow diagram of the experiment method

The potting soil for ginseng sprouts was purchased from Nongkyung (Jincheon-gun, Korea). The soil was composed of peat moss (70%, w/w) and perlite (30%, w/w). The potting soil treatment process is shown in Fig. 1. Potting soil (ca. 200 g) was placed in plastic beaker and treated using three different sterilization methods (non-sterilized, pasteurized at 60 °C for 30 min, and sterilized at 121 °C for 15 min) by autoclave (SJ-220A80, Sejong Technology Co. Ltd., Bucheon-si, Gyeonggi-do, Korea). Non-sterilized potting soil was used as the control. For potting soil inoculation, 20 mL suspensions of each strain were inoculated on potting soil (200 g) to reach a final concentration of ca. 10^6^–10^7^ CFU/g. The inoculated potting soil was shaken in a sterile plastic bag (Microgiene Co., Ltd, Suwon-si, Gyeonggi-do, Korea) for 10 min to mix it well. After mixing, potting soil (ca. 200 g) was packaged in a sterile plastic bag (Microgiene Co., Ltd). The packaged potting soil was stored in an incubator at 5, 15, 25, and 35 °C for 0, 3, 7, 10, 15, 30, and 60 days. These temperatures were chosen based on the recommended cultivation temperature of ginseng sprouts (25 °C), and low (5 and 15 °C) or high temperatures (35 °C) were chosen for accelerated testing.

### Measurement of pH and moisture content (%)

The pH level was measured using a pH meter (SevenEasy S20, Mettler Toledo, GmbH, Switzerland). The treated potting soil (1 g) was homogenized thoroughly with distilled water (20 mL). To determine weight loss, the samples were weighed using a digital balance (BCE224i-1SKR, Sartorius, Göttingen, Germany). The moisture content (%) of the potting soil was determined using the gravimetric method (Magdić et al., 2022). Moisture content (%) was calculated by the weight loss of the sample (50 g) maintained in a dry oven (DS-80-1, Dasol Scientific Co. Ltd., Hwaseong-si, Korea) at 105 °C until a constant weight was reached. The sample was allowed to cool in a desiccator before the potting soil weight was recorded.

### Bacterial enumeration

Potting soil samples (10 g) were homogenized with 90 mL of 0.2% peptone water (PW; Difco) in sterile stomacher bags (3 M) using a stomacher (BagMixer® 400, Interscience, France) for 90 s. After homogenization, aliquots (1 mL) of the sample were serially diluted tenfold with 9 mL of 0.2% PW. The diluent (0.1 mL) was plated onto each selective agar. Sorbitol MacConkey agar (CT-SMAC; Oxoid) supplemented with cefixime-tellurite (Microgiene Co., Ltd), Xylose Lysine Tergital-4 agar (XLT4, Oxoid), Baird-Parker agar (BPA, Oxoid) supplemented with egg yolk tellurite emulsion (Oxoid), and Mannitol Egg Yolk Polymyxin agar (MYP, Oxoid) supplemented with polymyxin B (Oxoid) and egg yolk emulsion (Oxoid) were used as selective agar to enumerate *E. coli* O157:H7, *S*. Typhimurium, *S. aureus*, and *B. cereus*, respectively. TSA containing 50 μg/mL rifampicin was used as a non-selective agar to count the total number of total viable cells including healthy and sublethally injured cells. All plates except for MYP (30 °C, 24–48 h) were incubated at 37 °C for 24–48 h, after which the typical colonies were enumerated.

### Statistical analysis

All experiments were repeated three times with duplicate samples. Before analysis, the means of the plate counts from three replicates were converted to log CFU/g. Analysis of variance (ANOVA) for a completely randomized design was performed using SPSS (version 25, IBM Corporation, Endicott, NY, USA). Means were separated using Duncan’s multiple range test when there were significant differences (*p* < 0.05) among treatments.

## Results and discussion

### pH and moisture content (%) in potting soil

The changes in pH and moisture content (%) in the potting soil by the three different sterilization methods are shown in Table 1. The pH levels of the non-sterilized, pasteurized, and sterilized potting soils were 5.76, 5.39, and 5.01, respectively. pH levels slightly decreased in the potting soil depending on the sterilization method. Tanaka et al. (2003) reported that the physicochemical properties of soil, such as pH, change after autoclaving. This change may be due to the release of organic acids from organic matter in the soil (Berns et al., 2008; Skipper and Westermann, 1973). However, it seems that pH levels of samples did not affect the populations of pathogenic bacteria. For example, non-sterilized potting soil was effective at decreasing levels of *Staphylococcus aureus* however, its pH level (5.76 ± 0.05) was higher than that of the pasteurized potting soil that were less effective. The moisture content (%) of potting soil did not significantly change with different sterilization methods (*p* > 0.05).

**Table 1:** pH and moisture content (%) of potting soil by three different sterilization methods

Sterilization methods	pH	Moisture content (%)
Non-sterilized potting soil	5.76 ± 0.05^A^	31.95 ± 0.06^A^
Pasteurized potting soil	5.39 ± 0.04^B^	31.92 ± 0.38^A^
Sterilized potting soil	5.01 ± 0.06^C^	31.31 ± 0.74^A^

^A–C^Means with the same uppercase letters in the same column are not significantly different (*p* < 0.05)

### Survival characteristics of pathogenic bacteria in potting soil by three different sterilization methods

**Fig. 2:**
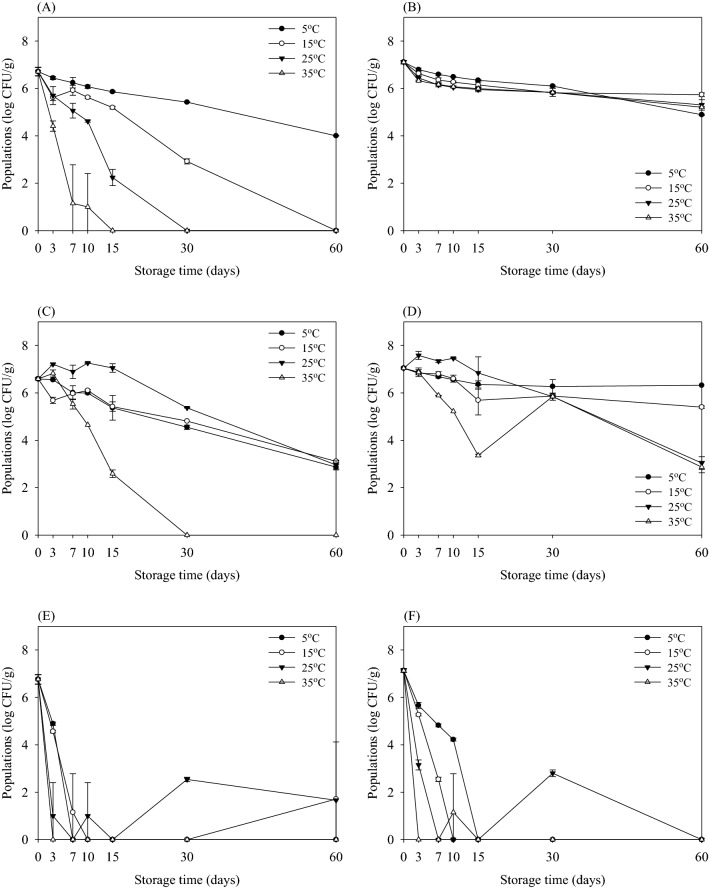
Survival (log CFU/g) of *Escherichia coli* O157:H7 in potting soil according to sterilization methods during storage at 5 °C (filled circles), 15 °C (open circles), 25 °C (down pointing triangles), and 35 °C (up pointing triangles) for 60 days when enumerated with selective agar (**A**, **C**, **E**) and non-selective agar (**B**, **D**, **F**). Sterilization methods using non-sterilized (**A**) and (**B**); pasteurized (**C**) and (**D**); and sterilized (**E**) and (**F**). Data represent mean ± standard deviations of three measurements

**Fig. 3:**
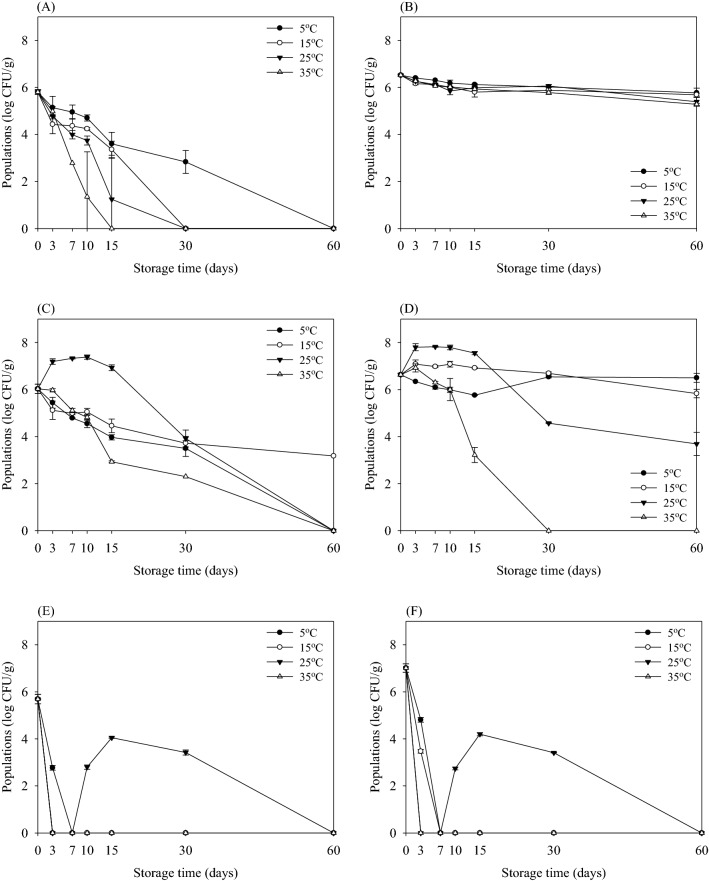
Survival (log CFU/g) of *Salmonella *Typhimurium in potting soil according to sterilization methods during storage at 5 °C (filled circles), 15 °C (open circles), 25 °C (down pointing triangles), and 35 °C (up pointing triangles) for 60 days when enumerated with selective agar (**A**, **C**, **E**) and non-selective agar (**B**, **D**, **F**). Sterilization methods using non-sterilized (**A**) and (**B**); pasteurized (**C**) and (**D**); and sterilized (**E**) and (**F**). Data represent mean ± standard deviations of three measurements

**Fig. 4:**
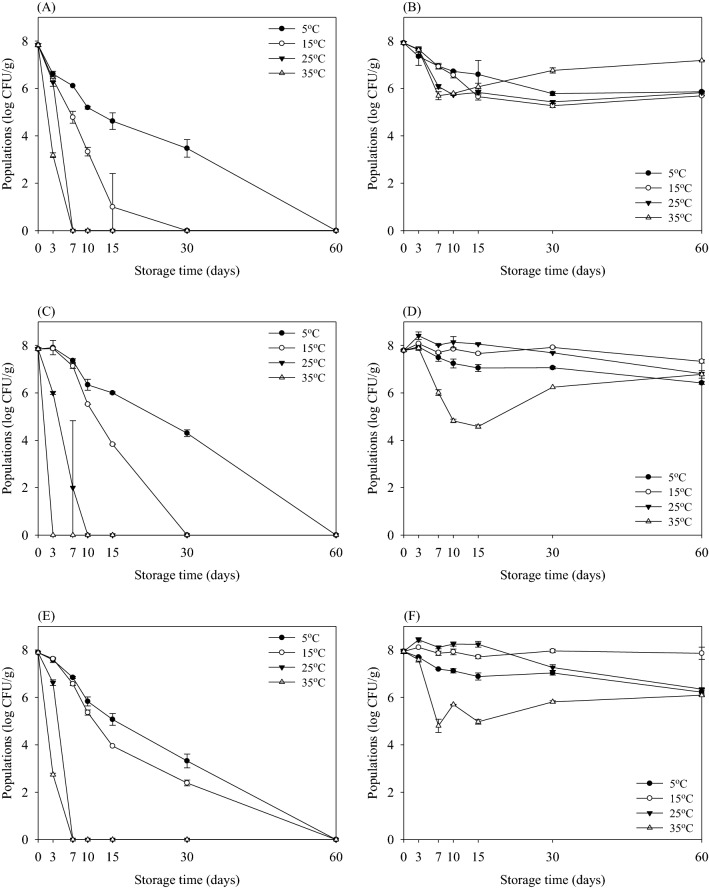
Survival (log CFU/g) of *Staphylococcus aureus* in potting soil according to sterilization methods during storage at 5 °C (filled circles), 15 °C (open circles), 25 °C (down pointing triangles), and 35 °C (up pointing triangles) for 60 days when enumerated with selective agar (**A**, **C**, **E**) and non-selective agar (**B**, **D**, **F**). Sterilization methods using non-sterilized (**A**) and (**B**); pasteurized (**C**) and (**D**); and sterilized (**E**) and (**F**). Data represent mean ± standard deviations of three measurements

**Fig. 5:**
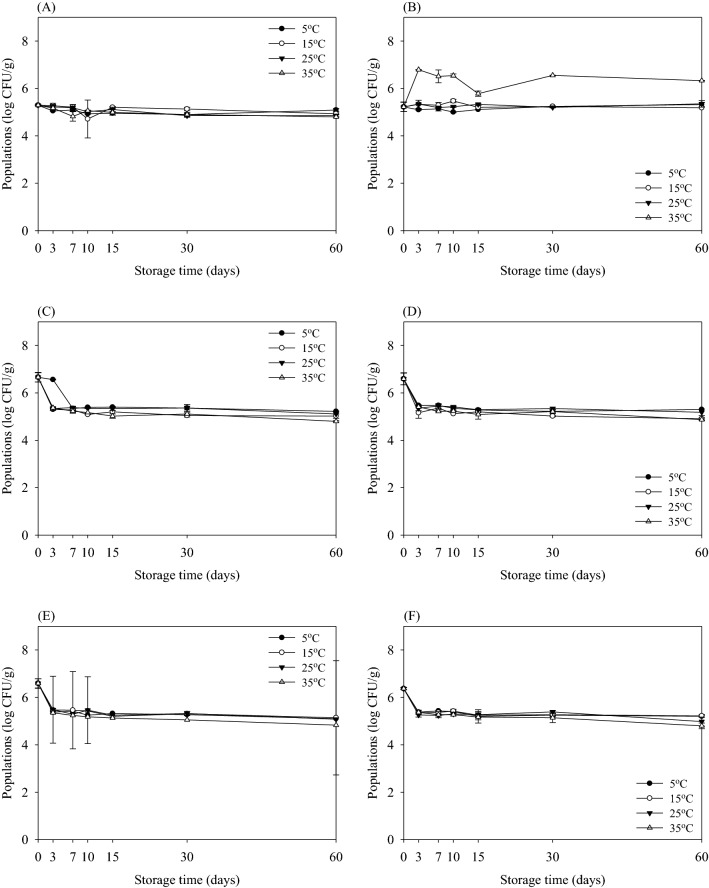
Survival (log CFU/g) of *Bacillus cereus* in potting soil according to sterilization methods during storage at 5 °C (filled circles), 15 °C (open circles), 25 °C (down pointing triangles), and 35 °C (up pointing triangles) for 60 days when enumerated with selective agar (**A**, **C**, **E**) and non-selective agar (**B**, **D**, **F**). Sterilization methods using non-sterilized (**A**) and (**B**); pasteurized (**C**) and (**D**); and sterilized (**E**) and (**F**). Data represent mean ± standard deviations of three measurements

The survival characteristics of *E. coli* O157:H7, *S*. Typhimurium, *S. aureus*, and *B. cereus* in potting soil treated using different sterilization methods during storage at various temperatures (5, 15, 25, and 35 °C) are shown in Figs. 2, 3, 4, and 5, respectively. The initial populations of *E. coli* O157:H7 in non-sterilized potting soil were 6.76 and 7.13 log CFU/g on selective (CT-SMAC) and non-selective agar (TSA), respectively (Fig. 2A, B). The population of *E. coli* O157:H7 in non-sterilized potting soil stored at 5 °C for 60 days was 4.00 log CFU/g (Fig. 2A). However, *E. coli* O157:H7 counts were reduced below detectable levels (≤ 2.00 log CFU/g) in non-sterilized potting soil after 60 days of storage at 15, 25, and 35 °C. In contrast, those stored at 5, 15, 25, and 35 °C were 4.89, 5.74, 5.30, and 5.21 log CFU/g, respectively, after 60 days when enumerated on TSA (Fig. 2B). These results indicate that the sublethal injury cell induces in non-sterilized potting soil. During 60 days of storage, the reduction in the population of *E. coli* O157:H7 in potting soil was higher at 35 °C than at 5 °C. In particular, the population of *E. coli* O157:H7 was reduced below detectable levels after storage for 30 days at 35 °C. In contrast, the population of *E. coli* O157:H7 was reduced to 2.87–3.11 log CFU/g at 5, 15, and 25 °C when enumerated on CT-SMAC (Fig. 2C). In addition, the population of *E. coli* O157:H7 was reduced to below detectable levels in sterilized potting soil after storage for 3 days at 35 °C (Fig. 2E). There were no appreciable differences between survived levels enumerated on CT-SMAC and TSA. According to these results, sterilized potting soil effectively reduced levels of *E. coli* O157:H7 without producing sublethally injured cells.

The survival curve of *S*. Typhimurium in potting soil treated with the three different sterilization methods is shown in Fig. 3. The initial populations of *S*. Typhimurium in non-sterilized potting soil were 5.80 and 6.52 log CFU/g as determined on selective (XLT4) and non-selective agar (TSA), respectively (Fig. 3A, B). Overall, the growth of *S*. Typhimurium was rapidly reduced compared to that of *E. coli* O157:H7 during storage for 60 days. At the end of the storage period, the populations of *S*. Typhimurium in pasteurized potting soil were reduced below detectable levels at 5, 25, and 35 °C (Fig. 3C). In contrast, *S*. Typhimurium in pasteurized potting soil was maintained at approximately 4.00 log CFU/g after storage at 15 °C for 60 days when enumerated on XLT4. Similar to *E. coli* O157:H7, *S*. Typhimurium was not detected in sterilized potting soil after storage for 3 days at 35 °C (Fig. 3E, F). There were no differences between survived levels enumerated on XLT4 and TSA. These results reveal that *S*. Typhimurium inactivated in sterilized potting soil without producing sublethally injured cells. In addition, populations of *S*. Typhimurium were not detected in sterilized potting soil after 7 days of storage at 5 and 15 °C. On the other hand, the population of *S*. Typhimurium decreased and then slightly increased in sterilized potting soil at 25 °C after 7 days. This phenomenon is probably a difference between survival mechanisms of bacteria in potting soil treated with non-sterilization and sterilization.

Figure 4 shows the populations of *S. aureus* in potting soil stored at various temperatures using three different sterilization methods. The initial populations of *S. aureus* in non-sterilized potting soil were 7.83 and 7.92 log CFU/g enumerated on selective (BPA) and non-selective agar (TSA), respectively (Fig. 4A, B). The initial population of *S. aureus* was maintained in three types of potting soil enumerated on TSA (Fig. 4B, D, F). On the other hand, the populations of *S. aureus* were reduced according to the storage temperature in the three types of potting soil during storage for 60 days at all temperatures, indicating that sublethal injury cell produced (Fig. 4A, C, E). Among the storage temperature, *S. aureus* was more effective at inhibiting at higher temperatures (25 and 35 °C) than at lower temperatures (5 and 15 °C). Similar to *E. coli* O157:H7 and *S*. Typhimurium, storage at 35 °C in pasteurized and sterilized potting soil was the most effective at reducing *S. aureus* after storage for 3 and 7 days, respectively. These results indicated that the survival of *E. coli* O157:H7, *S*. Typhimurium, and *S. aureus* depends on the storage temperature of the potting soil.

Among the pathogenic bacteria tested, *B. cereus* was the most resistant in the soil (Fig. 5). The initial populations of *B. cereus* in non-sterilized potting soil were 5.29 and 5.22 log CFU/g enumerated on selective (MYP) and non-selective agar (TSA), respectively (Fig. 5A, B). The initial population of *B. cereus* in non-sterilized potting soil did not change during storage for 60 days at all temperatures when enumerated on MYP (Fig. 5A), whereas the growth of *B. cereus* stored at 35 °C slightly increased on TSA (Fig. 5B). The results showed that the initial levels of *B. cereus* were maintained in non-sterilized potting soil during 60 days of storage at all temperatures when enumerated on MYP (Fig. 5A). On the other hand, the number of *B. cereus* in three types of potting soil grown on both TSA and MYP were almost similar, indicating that there was no injury produced.

The survival and growth of microorganisms in soil differ depending on various factors, including pH, temperature, moisture content, nutrient availability, and soil type (Alegbeleye et al., 2018). The storage temperature is important factor for the survival and growth of microorganisms in the soil (Arrus et al., 2006). In this study, storage at 35 °C was effective in reducing the levels of pathogenic bacteria in pasteurized and sterilized potting soils during storage. Several studies have demonstrated that higher temperatures may be more effective in inhibiting pathogenic bacteria than lower temperatures. Cools et al. (2001) observed that *E. coli* populations in sandy soil were reduced below detectable levels after 68 days at 5 °C, whereas populations of *E. coli* were reduced below detectable levels after 26 days of storage at 25 °C. García et al. (2010) showed that the survival of *S*. Typhimurium in the top soil was maintained at 5 °C compared to 25 °C. Similar studies reported by Danyluk et al. (2007) showed that the population of *Salmonella* spp. was reduced in soil from almond orchards by approximately 1.00 and 5.00 log CFU/g at 20 °C and 35 °C after 30 days of storage, respectively. This was probably due to the differences in soil moisture levels according to the storage temperature. A few studies have demonstrated that the populations of bacteria or viruses are reduced in dry environments rather than in cool and moist environments (Ghorbani et al., 2008; Santamaria and Toranzos, 2003). In addition, the population of pathogenic bacteria was more effective at inhibiting in sterilized potting soil than in non-sterilized and pasteurized potting soil. This was probably due to the absence of naturally resident microbiomes in the soil after sterilization. In other words, the growth of pathogenic bacteria increased without competitive organisms than that with competitive organisms. This phenomenon is consistent with the results of Choi et al. (2010), who reported that growth of *S. aureus* was more active without competitive organisms compared to with competitive organisms in bean sprouts. These results indicate the difference between survival mechanisms of bacteria in potting soil treated with non-sterilization and sterilization. In this study, the growth of *E. coli* O157:H7 and *B. cereus* tended to be maintained in non-sterilized and pasteurized potting soil at after 60 days of storage at 5 °C. Several studies have shown that populations of pathogenic bacteria are maintained in the soil during plant cultivation. Zhang et al. (2009) demonstrated that *E. coli* O157:H7 survived in soil for at least 60 days during storage at 23 °C for 12 h (day) and 7 °C for 12 h (night). Jechalke et al. (2019) reported that the average population of *S*. Typhimurium persisted at approximately 2.50 log CFU/g in the soil during the growth period of lettuce (44 days). Another study showed that the population of *Salmonella* spp. increased by 2.50 log CFU/tomato on tomatoes in contact with soil during storage at 20 °C for 4 days and remained constant for an additional 10 days (Guo et al., 2002). These microorganisms may cause the soil on which plants grow is a potential source of contamination. Soil inoculated with *E. coli* was detected on the surface of spinach after cultivation for 42 days at 20–26 °C (Warriner et al., 2003). Ingham et al. (2004) showed that *E. coli* was detected in lettuce grown in soil in the presence of bovine manure contaminated with *E. coli*, indicating that *E. coli* in soil was transferred to lettuce. According to this result, the survival of *B. cereus* was not influenced by the storage temperature or sterilization method. *B. cereus* is widely distributed in natural environments, including soil and aquatic habitats, from which it can be transferred to plants and food products (Bartoszewicz and Czyżewska, 2017). In particular, the soil is the primary source of contamination in raw vegetables contaminated with *B. cereus* (Kotiranta et al., 2000). In our previous study, *B. cereus* was frequently detected in raw materials (ginseng sprouts, ginseng seedlings, potting soil, and green moss) and working tools (gloves, working table, dishcloth, scissors, boots, and knob of greenhouses) in ginseng sprout farms (data not shown). These results indicated that *B. cereus* contaminated with soil could be easily transferred to various raw materials and working tools on farms. Therefore, appropriate technologies to control the contamination of *B. cereus* in potting soil need to be developed. Furthermore, further study is needed to understand the difference in inactivation susceptibility according to bacterial strains.

These results could provide valuable information for improving the microbial safety of reused soil using appropriate sterilization methods and storage temperatures. However, limited studies have investigated the effects of sterilization methods and storage temperature on the survival of pathogenic bacteria in the soil. Our results showed that storage temperature is one of the most important factors for reusing potting soil. Moreover, sterilization methods, such as pasteurization or sterilization in soil, are effective treatments for reducing the survival of pathogenic bacteria. Therefore, storage at high temperatures after pasteurization or sterilization of potting soil can be used to effectively control the presence of pathogenic bacteria. Further studies should be conducted to investigate the appropriate conditions, such as sterilization temperature and time, to control the microbial safety and physiochemical characteristics of potting soil.
